# Biofilm-Forming Clinical *Staphylococcus* Isolates Harbor Horizontal Transfer and Antibiotic Resistance Genes

**DOI:** 10.3389/fmicb.2017.02018

**Published:** 2017-10-16

**Authors:** Sandra Águila-Arcos, Itxaso Álvarez-Rodríguez, Olatz Garaiyurrebaso, Carlos Garbisu, Elisabeth Grohmann, Itziar Alkorta

**Affiliations:** ^1^Instituto Biofisika (UPV/EHU, CSIC), Department of Biochemistry and Molecular Biology, University of the Basque Country, Bilbao, Spain; ^2^Department of Conservation of Natural Resources, Soil Microbial Ecology Group, NEIKER-Tecnalia, Derio, Spain; ^3^Life Sciences and Technology, Beuth University of Applied Sciences, Berlin, Germany

**Keywords:** Staphylococci, biofilm, relaxases, antibiotic resistance, nosocomial infections

## Abstract

Infections caused by staphylococci represent a medical concern, especially when related to biofilms located in implanted medical devices, such as prostheses and catheters. Unfortunately, their frequent resistance to high doses of antibiotics makes the treatment of these infections a difficult task. Moreover, biofilms represent a hot spot for horizontal gene transfer (HGT) by bacterial conjugation. In this work, 25 biofilm-forming clinical staphylococcal isolates were studied. We found that *Staphylococcus epidermidis* isolates showed a higher biofilm-forming capacity than *Staphylococcus aureus* isolates. Additionally, horizontal transfer and relaxase genes of two common staphylococcal plasmids, pSK41 and pT181, were detected in all isolates. In terms of antibiotic resistance genes, *aac6-aph2a, ermC*, and *tetK* genes, which confer resistance to gentamicin, erythromycin, and tetracycline, respectively, were the most prevalent. The horizontal transfer and antibiotic resistance genes harbored on these staphylococcal clinical strains isolated from biofilms located in implanted medical devices points to the potential risk of the development and dissemination of multiresistant bacteria.

## Introduction

Staphylococci, mainly *Staphylococcus aureus* and *Staphylococcus epidermidis*, are well-known causative agents of a large number of human infectious diseases, including skin, soft tissue, respiratory tract, bone, joint and endovascular infections, as well as infections related to implanted medical devices (Otto, 2012; Le et al., 2014). Their pathogenicity is due not only to the virulence factors that they express, but also to the ability of these bacteria to form biofilms (i.e., deeply seated microbial communities attached to inert or living surfaces; Costerton et al., 1999; Otto, 2008). The treatment of biofilm-associated infections is considered a challenging task owing to their inherent resistance to (i) antimicrobial agents and (ii) the host immune system (Hoiby et al., 2010). Moreover, nowadays, the incidence of antibiotic resistant pathogenic bacteria in clinical settings is dramatically increasing, making treatment of bacterial infections one of our most serious health threats (Guridi et al., 2015). This problem arises from the resistance phenotype of bacteria that harbor resistance genes in their chromosomal and/or plasmid DNA.

Bacteria can acquire resistance genes by horizontal gene transfer (HGT). Actually, conjugative plasmid-mediated HGT is considered the most important process in the emergence of new resistant pathogens (Schiwon et al., 2013). It is well-documented that bacterial conjugation can occur within biofilms since they provide an ideal situation for the exchange of genetic material of various origins (Christensen et al., 1998; Hausner and Wuertz, 1999). On the other hand, bacterial conjugation can induce biofilm formation since the cell-to-cell contact established for gene exchange favors the close proximity of bacteria required for biofilm formation (Ghigo, 2001; Molin and Tolker-Nielsen, 2003; Reisner et al., 2006; Yang et al., 2008; D'Alvise et al., 2010). This link between biofilms and bacterial conjugation increases both the risk of biofilm-related infections and the conjugative spread of virulence factors.

In this work, we studied 25 staphylococcal biofilm-forming clinical isolates belonging to the following species: *S. aureus, S. epidermidis, S. hominis*, and *S. capitis*. These species are commonly found on human skin and can cause biofilm-forming healthcare-associated infections. Both horizontal transfer and antibiotic resistance genes were detected in these staphylococcal clinical isolates. This work adds valuable information on the risk of development and dissemination of antibiotic resistance in *Staphylococcus* biofilm-forming clinical isolates.

## Materials and methods

### Bacterial strains

A total of 25 staphylococcal biofilm-forming clinical isolates were kindly provided by Hospital Universitario Donostia, Spain. In addition, they provided data on their antibiotic resistance phenotype, determined by diffusion discs on agar. The origin and antibiotic resistance phenotype of each isolate are shown in Table 1.

**Table 1:** Origin and antibiotic resistance phenotype of the Staphylococcal biofilm-forming clinical isolates used in this work.

**No**.	**Isolate**	**Origin**	**Antibiotic resistance^a^^,^^b^**
1	*S. aureus* 312042	Prosthesis	AMX, AMC, CFZ, CLI, CLOX, ERY, LVX
2	*S. aureus* 410099	Prosthesis	AMX, AMC, CFZ, CLOX, GEN, LVX, MUP
3	*S. aureus* 218154	Prosthesis	AMX, AMC, CFZ, CLI, CLOX, ERY, LVX, MUP, RIF
4	*S. aureus* 339031	Catheter	AMX, AMC, CFZ, CLI, CLOX, ERY, LVZ
5	*S. aureus* 215642	Prosthesis	AMX
6	*S. epidermidis* 213303	Prosthesis	AMX, AMC, CFZ, CLI, CLOX, CTX, ERY, GEN, LVX, MUP, RIF
7	*S. hominis* 313732	Prosthesis	AMX, AMC, CFZ, CLI, CLOX, CTX, ERY, GEN, LVX, MUP
8	*S. capitis* 316479	Prosthesis	AMX, AMC, CFZ, CLOX, GEN, LVX, MUP
9	*S. epidermidis* 319622	Prosthesis	AMX, ERY, TET
10	*S. epidermidis* 219691	Prosthesis	AMX, ERY, LVX, MUP, RIF
11	*S. aureus* 214967	Ulcer	AMX, MUP
12	*S. epidermidis* 239879	Catheter	AMX, AMC, CFZ, CLI, CLOX, ERY, GEN, MUP, TET
13	*S. epidermidis* 239891	Catheter	AMX, AMC, CFZ, CLOX, GEN, LVX, MUP, RIF
14	*S. aureus* 337423-1	Catheter	AMX, AMC, CFZ, CLOX, LVX
15	*S. aureus* 338550-1	Catheter	AMX, AMC, CFZ, CLOX, ERY, LVX
16	*S. aureus* 339031-2	Catheter	AMX, AMC, CFZ, CLOX, ERY, LVX
17	*S. aureus* 339056-2	Catheter	AMX, AMC, CFZ, CLOX, ERY, LVX
18	*S. aureus* 339300	Catheter	AMX, AMC, CFZ, CLOX, LVX
19	*S. aureus* 338503	Catheter	AMX
20	*S. epidermidis* 214627-A	Articular fluid from patient with prosthesis	AMX, AMC, CFZ, CLI, CLOX, CTX, ERY, GEN, LVX, MUP, RIF
21	*S. epidermidis* 310301-1	Articular fluid from patient with prosthesis	AMX, AMC, CFZ, CLI, CLOX, CTX, ERY, GEN, LVX, MUP, RIF
22	*S. epidermidis* 338400-1	Catheter	AMX, AMC, CFZ, CLOX, MUP
23	*S. epidermidis* 338515-1	Catheter	AMX, AMC, CFZ, CLI, CLOX, CTX, ERY, GEN, MUP, RIF
24	*S. epidermidis* 338684	Catheter	AMX, AMC, CFZ, CLI, CLOX, CTX, GEN, LVX, MUP, RIF
25	*S. epidermidis* 216663	Articular fluid from patient with prosthesis	AMX, AMC, CFZ, CLI, CLOX, ERY, GEN, RIF

a *Resistance to antibiotics was analyzed by diffusion discs on agar by Hospital Universitario Donostia*.

b *AMX, amoxicillin; AMC, amoxicillin + clavulanic acid; CFZ, cefazolin; CLI, clindamycin; CLOX, cloxacillin; CTX, cotrimoxazol; ERY, erythromycin; GEN, gentamicin; LVX, levofloxacin; MUP, mupirocin; RIF, rifampicin; TET, tetracycline; VAN, vancomycin*.

### Growth conditions

Swabs from the clinical isolates were plated on tryptic soy agar (TSA) and incubated at 37°C. Subsequently, a single colony of each isolate was grown in 10 ml of tryptic soy broth (TSB) supplemented with at least two antibiotics to which the strain was phenotypically resistant (see Table 1), at 37°C overnight. The culture was centrifuged at 8,000 × g for 10 min. Then, the pellet was resuspended in 2 ml of TSB medium containing 40% (v/v) glycerol and stored at −80°C.

For this study, strains were grown in TSB medium at 37°C and 200 rpm. TSB medium and TSA plates were supplemented, when required, with amoxicillin (8 μg/ml), cloxacillin [2 μg/ml for *S. aureus* and 0.5 μg/ml for coagulase negative staphylococci (CoNS)], erythromycin (4 μg/ml), mupirocin (520 μg/ml), tetracycline (8 μg/ml), gentamicin (20 μg/ml), rifampicin (2 μg/ml), or levofloxacin (2 μg/ml).

### DNA extraction

Plasmid DNA was extracted from the 25 clinical isolates with the ATP^TM^ Plasmid Midi kit (ATP biotech Inc., Taiwan), according to the manufacturer's instructions.

### Detection of small plasmids by agarose gel electrophoresis

To detect small plasmids (molecular size < 20 kb), 1 μg of total extracted plasmid DNA was linearized by incubation with 30 U of *Aspergillus oryzae* nuclease S1 (Sigma, Spain) at 37°C for 45 min. Nuclease S1 cuts one strand of the DNA at the nick site and its activity results in linearized plasmids (Germond et al., 1974). Different enzyme concentrations were studied to optimize nuclease S1 digestion (data not shown). Linearized plasmids were visualized on 1% (w/v) agarose gels in 1 × TAE buffer.

### Detection of large plasmids by pulsed field gel electrophoresis

Detection of large plasmids (molecular size > 20 kb) was carried out by Pulsed Field Gel Electrophoresis (PFGE) as described by Barton et al. (1995) with modifications. Bacteria were grown in 2 ml of TSB medium overnight at 37°C and 200 rpm. Cultures were diluted in PIV buffer [10 mM Tris-HCl (pH 8), 1 M NaCl] until OD_600_ = 1. Then, 600 μl of diluted culture were centrifuged at 11,000 × g for 2 min. Subsequently, the pellet was washed with 500 μl of PIV buffer and centrifuged again. The pellet was resuspended in 300 μl of PIV buffer and incubated at 42°C for 10 min. Next, 150 μl of the sample were mixed with 150 μl of 2% (w/v) low-melting agarose (BioRad) which had been preincubated at 42°C. The mixture was transferred into the plugs, incubated at room temperature for 10 min and, subsequently, for 15 min at 4°C. Once solidified, gel plugs were incubated at 37°C for 5–6 h with shaking (600 rpm) in 1 ml of lysis buffer EC [6 mM Tris-HCl (pH 8), 1 M NaCl, 100 mM EDTA (pH 8), 0.2% (w/v) sodium deoxycholate, 0.5% (w/v) n-lauroylsarcosine, 100 μg/ml lysozyme, 50 μg/ml lysostaphin]. After cell lysis, gel plugs were transferred to new tubes containing 1 ml of EPS solution [1% (w/v) n-lauroylsarcosine, 0.5 M EDTA (pH 8), 100 μg/ml proteinase K] and then incubated at 56°C for 16–20 h. Next, five washes with 1 ml of TE buffer [10 mM Tris-HCl (pH 8), 1 mM EDTA (pH 8)] at 50°C for 30 min each were carried out. For nuclease S1 digestion, each gel plug was cut into two slices. Each slice was incubated twice in 100 μl of digestion solution [50 mM NaCl, 30 mM sodium acetate (pH 4.5), 5 mM ZnSO_4_] at room temperature for 15 min. Then, slices were incubated at 37°C for 45 min with 1 U of *A. oryzae* nuclease S1 (Sigma) in 100 μl of digestion solution. The reaction was stopped by transferring the slices to 1 ml of TE buffer for 1 h. Digested slices were applied to wells in 1% (w/v) Pulsed Field Certified Agarose (BioRad) prepared in 0.5 × TBE buffer [45 mM Tris (pH 8), 45 mM boric acid, 1 mM EDTA] and run in CHEF-DR® III System (BioRad) at 6 V/cm, a field angle of 120°, and switch times of 5 to 35 s for 22 h. Lambda Ladder PFGE (New England Biolabs, Ispwich, U.S) was used as molecular size marker and pSK41 plasmid (46.4 kb) was used as positive control. Gels were stained with GelRed Nucleic Acid Stain (Biogen Científica, Madrid, Spain). Bands were visualized by ChemiDoc XRS System (BioRad). Images were analyzed by Quantity One 1-D Analysis Software (BioRad).

### Polymerase chain reaction (PCR) and southern blotting

PCR and Southern blotting assays, specific for horizontal transfer and antibiotic resistance genes, were performed using the strains and plasmids indicated in Table 2 as reference DNA. Oligonucleotides used for gene detection are listed in Table 3. Each 25 μl PCR reaction mixture contained 1.25 U Taq polymerase (New England Biolabs, Ipswich, U.S.), 1 × PCR buffer, 0.5 μM of each primer, 0.2 mM deoxynucleoside triphosphates and 20 ng of template DNA (plasmid DNA). Amplifications were carried out in a C1000™ Thermal Cycler (BioRad). PCR temperature profiles are shown in Table 4. PCR products were separated by agarose gel electrophoresis, transferred to a membrane (Sambrook and Russel, 2001), and then hybridized with the corresponding specific DIG-labeled probe using the PCR DIG Probe Synthesis Kit (Roche, Mannheim, Germany). Detection of DNA sequences was performed with the DIG Luminescent Detection Kit (Roche) according to the manufacturer's instructions.

**Table 2:** Bacterial strains and plasmids used as reference for PCR and Southern blotting.

**Strain**	**Characteristics**	**References**
***Bacillus subtilis***		
BD662	pBD90, *ermD*	Gryczan et al., 1984
BD1156	pBD370, *ermG*	Monod et al., 1987
***Enterococcus faecalis***		
RE25	pRE25, *ermB, tetM*	Schwarz et al., 2001
V583	pTEF1, pTEF2, pTEF3, *vanB*	Paulsen et al., 2003
***S. aureus***		
RN3259	pT181, *tetK, pre*_pT181_	Khan et al., 1981
SK5428	pSK41, *acc(6′)-Ie-aph(2′)-Ia*, tra^+^, *pre*_pSK41_, *nes*_pSK41_	Firth et al., 1993
***S. haemolyticus***		
VPS617	*tetK, ermC*	Perreten et al., 2005

**Table 3:** Oligonucleotides used for the detection of antibiotic resistance and transfer genes.

**Gene**	**Oligonucleotide**	**Sequence (5′ 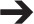 3′)**	**Acc. No^a^**	**Nucleotide position**	**Reference**
**Antibiotic resistance genes**	*aac6-aph2a* fw *aac6-aph2a* rev	GCCAGAACATGAATTACACGAG CTGTTGTTGCATTTAGTCTTTCC	NC_005024	42,981–43,002 43,569–43,591	Schiwon et al., 2013
	*ermB* fw *ermB* rev	GCATTTAACGACGAAACTGGCT GACAATACTTGCTCATAAGTAATGGT	U00453	6,796–6,817 7,343–7,368	Böckelmann et al., 2009
	*ermC* fw *ermC* rev	CGTAACTGCCATTGAAATAGACC TCCTGCATGTTTTAAGGAATTG	V01278	2,555–2,577 2,079–2,100	Schiwon et al., 2013
	*ermD* fw *ermD* rev	CGGGCAAATATTAGCATAGACG ATTCTGACCATTGCCGAGTC	M29832	544–565 988–1,007	Schiwon et al., 2013
	*ermG* fw *ermG* rev	TGCAGGGAAAGGTCATTTTAC AACCCATTTCATTACAAAAGTTTC	M15332	785–805 1,245–1,268	Schiwon et al., 2013
	*tetK* fw *tetK* rev	TTTGAGCTGTCTTGGTTCATTG AGCCCACCAGAAAACAAACC	CP000045	1,398–1,419 1,918–1,937	Schiwon et al., 2013
	*tetM* fw *tetM* rev	GAACTCGAACAAGAGGAAAGC ATGGAAGCCCAGAAAGGAT	M85225	1,114–1,134 1,835–1,853	Tenover and Rasheed, 2004
	*vanB* fw *vanB* rev	CCCGAATTTCAAATGATTGAAAA CGCCATCCTCCTGCAAAA	L06138	113–135 552–569	Miele et al., 1995
**Transfer genes**	pSK41 *pre* fw pSK41 *pre* rev	CTGGACTAAAAGGCATGCAA GCAGTTTTCCATCACGCATA	AF051917	20,674–20,693 20,298–20,317	Schiwon et al., 2013
	pSK41 *nes* fw pSK41 *nes* rev	AGCGCTAGTAGGATTAAAG CATAATAAATGTGCGTGAGG	AF051917	10,016–10,034 9,706–9,725	Schiwon et al., 2013
	pT181 *pre* fw pT181 *pre* rev	TCGAACAGAATTATACAGGCAA CTGACTTATTTGCTCATGTTTAGC	CP000045	2,708–2,729 3,082–3,105	Schiwon et al., 2013
	*traE* pSK41 fw *traE* pSK41 rev	TATCATTGATCC(T/C)GAA(A/G)ATGAAT TCTTTTGT(T/G)ATTTCGTCCCATAA	AF051917	27,456–27,478 28,060–28,082	Schiwon et al., 2013
	*traG* pSK41 fw *traG* pSK41 rev	GTGTTGACGGTTCGGGTATC TTTTCCGTCTGAACCTCCAC	AF051917	30,132–30,151 30,570–30,589	Schiwon et al., 2013
	*traK* pSK41 fw *traK* pSK41 rev	TATCTAAAGACCACCCAGCTAGAG TACTTGTTTCAAACTCTACAGTAGC	AF051917	34,636–34,660 35,185–35209	Schiwon et al., 2013
	*traL* pSK41 fw *traL* pSK41 rev	ATGGGGACTATGGCAGGTAG AAGTTTTGCACCACTTCCAG	AF051917	36,279–36,298 36,667–36686	Schiwon et al., 2013
	*traM* pSK41 fw *traM* pSK41 rev	TGTTGTATGGGGAAAACAAGC GCTGGGCTTATAGC(A/G)TCATC	AF051917	36,870–36,890 37,051–37,070	Schiwon et al., 2013

a *Accession Number from Gene Bank*.

**Table 4:** PCR conditions.

**Genes amplified**	**Denaturation**	**Primer annealing**	**Elongation**
*aac6-aph2a, ermD, ermG*,	95°C, 30 s	55°C, 30 s	72°C, 30 s
*ermC, tetK, tetM*	95°C, 30 s	58°C, 30 s	72°C, 30 s
*ermB, vanB*	95°C, 30 s	60°C, 45 s	72°C, 60 s
*traE, traG, traK, traL, traM, pre_pT181_*	95°C, 30 s	55°C, 45 s	72°C, 60 s
*pre_pSK41_, nes_pSK41_*	95°C, 60 s	50°C, 60 s	72°C, 120 s

*An initial denaturation step was performed, consisting of 2 min at 95°C, except for pre_pSK41_, nes_pSK41_, that were denaturated for 4 min. Then, 30 cycles consisting of denaturation, primer annealing and elongation steps were performed at the conditions (temperature and time) specified. A final elongation step at 72°C was performed during 5 min, except for pre_pSK41_, nes_pSK41_ in which it lasted 10 min*.

### Biofilm formation

To test the 25 clinical isolates for biofilm formation, a quantitative adherence assay (Christensen et al., 1985) with some modifications was used. Briefly, 200 μl of TSB medium in 96-well flat-bottom polystyrene plates were inoculated with 10 μl overnight bacterial cultures and grown without shaking at 37°C for 24 h. Planktonic bacteria were removed from each well. Then, three washes with distilled water per well were carried out. Next, 125 μl of 0.1% (w/v) crystal violet solution were added to each well and incubated for 10 min at room temperature. Subsequently, three washes with distilled water were again performed. To solubilize the dye, 200 μl of 33% (v/v) glacial acetic acid solution were added to each stained well and incubated for 10 min at room temperature. TSB medium was used as negative control. The optical density of the attached bacteria was measured in a microplate reader at 570 nm (in triplicate for each strain). The ability to form biofilm was attributed as: OD_570_ < 0.120, no biofilm-forming; 0.120 < OD_570_ < 0.240, weak biofilm-forming; OD_570_ > 0.240, strong biofilm-forming (Christensen et al., 1985; Di Rosa et al., 2006); and OD_570_ > 1.5, very strong biofilm-forming. Dilutions were performed when absorbance values were higher than the limit of accurate detection. To classify the isolates into significant groups, statistical analysis was performed using SigmaPlot program and Student's *t*-test or Mann–Whitney *U*-test.

## Results

### All clinical isolates, except one, harbored plasmids

**Figure 1:**
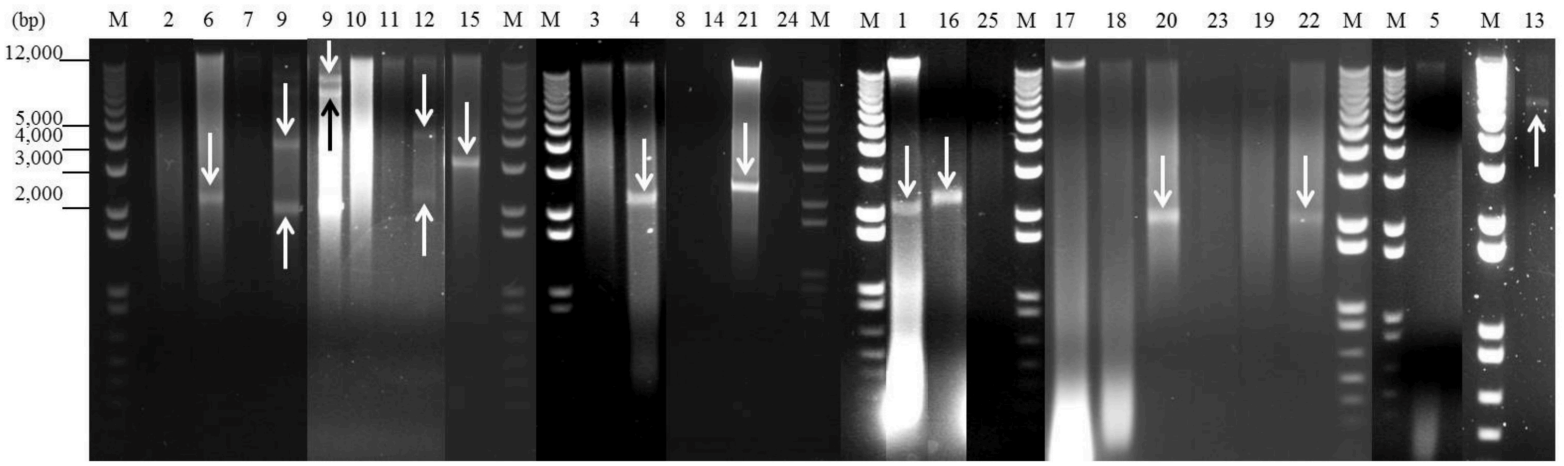
Detection of plasmids from 25 staphylococcal clinical isolates by agarose gel electrophoresis after digestion with nuclease S1. One microgram of plasmid DNA from each isolate was digested with 30 U of nuclease S1 at 37°C for 45 min. After digestion, the plasmids were analyzed by 1% (w/v) agarose gel electrophoresis in 1 × TAE buffer. Lanes 1–25: digested plasmid DNA from each strain (lane numbers correspond to the number of the isolate). Lanes M: DNA molecular weight marker 1 kb Plus DNA Ladder. Bands corresponding to plasmids are indicated with arrows.

**Figure 2:**
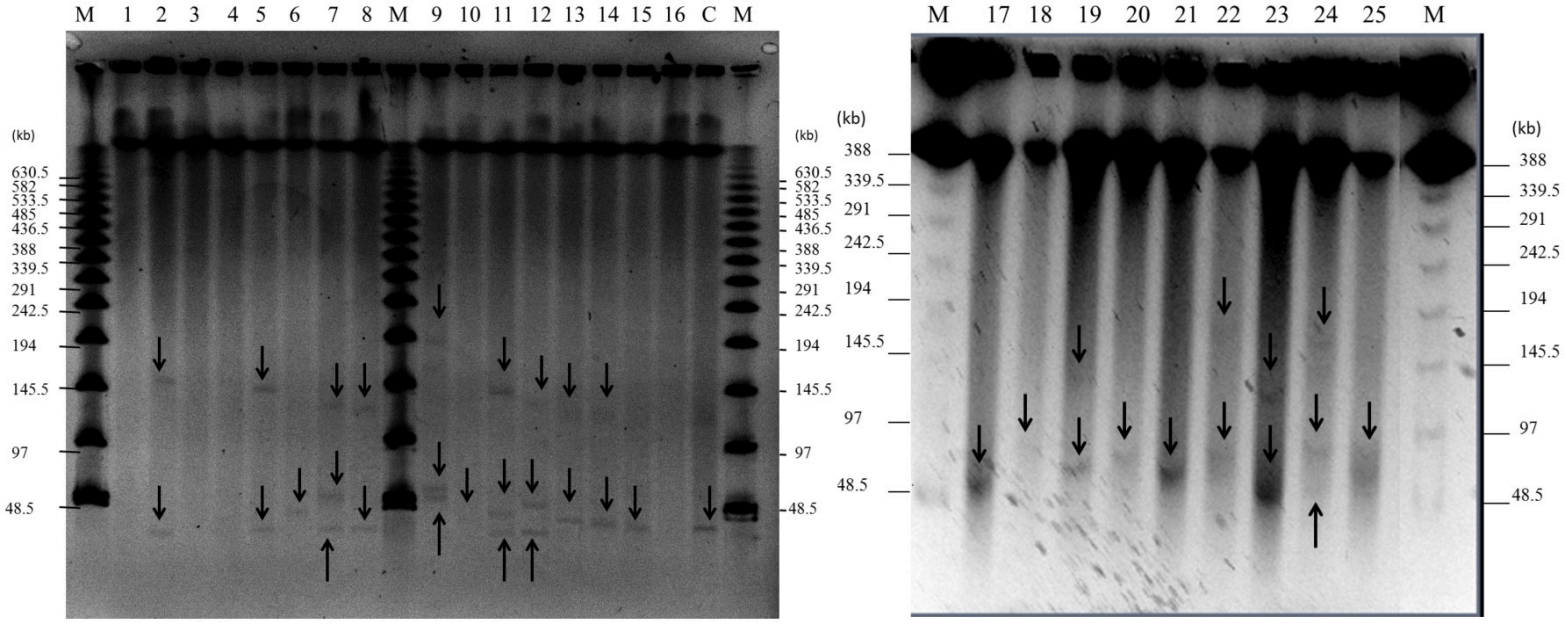
Detection of plasmids in 25 staphylococcal clinical isolates by PFGE. Large plasmids (>30 kb) were analyzed by PFGE after digestion with nuclease S1 at 37°C for 45 min. Lanes 1–25: clinical isolates; lane number corresponds to the number of the isolate. Lane C: positive control, plasmid pSK41 (46.4 kb) extracted from SK5428 strain. Lane M: Lambda Ladder PFGE molecular size marker. Arrows point to detected plasmids.

Plasmid DNA was extracted from the 25 clinical isolates and then analyzed by agarose gel electrophoresis and PFGE (Figures 1, 2). Since plasmid DNA samples are sometimes contaminated with chromosomal DNA, as suggested in Figure 1 for some of our isolates (i.e., 1, 3, 17, 21), after the extraction of plasmid DNA, we decided to test for such contamination. To this purpose, 16S rRNA from isolates 1, 3, 17, and 21 was amplified by PCR as explained in Broszat et al. (2014). The obtained amplicons were analyzed by 1% (w/v) agarose gel electrophoresis in 1 × TAE buffer. As observed in Supplementary Figure 1, some of our plasmid DNA samples appear to be contaminated with chromosomal DNA. Nonetheless, as reflected in Figures 1, 2, the majority of the extracted DNA corresponds to plasmid DNA.

As shown in Figures 1, 2 and Table 5, a total of 54 plasmids of sizes ranging from 2 to 200 kb were detected using both methods: 15 small plasmids (size < 20 kb) and 39 large plasmids (size > 20 kb; Shearer et al., 2011). All clinical isolates contained at least one plasmid, except isolate 3. The combination of agarose gel electrophoresis and PFGE is unable to detect plasmids between 13 and 45 kb. Then, *a priori*, our clinical isolates could harbor more plasmids than observed here. In particular, isolate 3 could harbor a plasmid between 13 and 45 kb, which could explain the apparent lack of plasmid observed for this isolate.

**Table 5:** Antibiotic resistance profiles, transfer genes, plasmid content, and biofilm-forming capacity of staphylococcal clinical isolates.

**Isolate**	**Antibiotic resistance phenotype^a^**	**Antibiotic resistance genotype^b^**	**Transfer genes**	**Plasmids^c^**		**Biofilm-forming capacity 24 h^d^**
				**<20 kb**	**>20 kb**	
1	ERY	*ermB, ermC, tetK, aac6-aph2a*	*pre*_pSK41_, *nes*_pSK41_, *pre*_pT181_, traE, traG, traK, traL, traM^*^	1	0	0
2	GEN	*ermB, ermC, ermG, tetK, aac6-aph2a*	*pre*_pSK41_, *pre*_pT181_, *traG, traL*	0	2	1
3	ERY	*ermC, tetK, aac6-aph2a*	*pre*_pSK41_, *pre*_pT181_, traL^*^	0	0	1
4	ERY	ermB^*^, *ermC, tetK, aac6-aph2a*	*pre*_pSK41_, *nes*_pSK41_, *pre*_pT181_, *traL*	1	0	0
5	–	ermB, ermC, tetK^*^, aac6-aph2a^*^	*pre*_pSK41_, *pre*_pT181_, *traK, traL*	0	2	3
6	ERY, GEN	*ermB, ermC, tetK, aac6-aph2a*	*pre*_pSK41_, *pre*_pT181_, *traG, traK, traL*	1	1	2
7	ERY, GEN	*ermB, ermC, tetK, aac6-aph2a*	*pre*_pSK41_, *nes*_pSK41_, *pre*_pT181_, *traE, traK, traL*	0	3	2
8	GEN	ermC, tetK^*^, *aac6-aph2a*	*pre*_pSK41_, *nes*_pSK41_, *pre*_pT181_, *traG, traK*	0	2	3
9	ERY, TET	ermC, tetK^*^	*pre*_pSK41_^*^, *pre*_pT181_, traL^*^	4	3	2
10	ERY	*ermB, ermC, tetK, aac6-aph2a, vanB*	*pre*_pSK41_, *pre*_pT181_, *traG, traL*	0	1	2
11	–	*ermB, ermC, tetK*	*pre*_pSK41_, *nes*_pSK41_, *pre*_pT181_, *traE, traG, traL, traM*	0	3	1
12	ERY, GEN, TET	*ermC, tetK, aac6-aph2a*	*pre*_pSK41_^*^, *pre*_pT181_, traG^*^, *traL*	2	3	3
13	GEN	*ermB, ermC, tetK, aac6-aph2a*	*pre*_pSK41_, *pre*_pT181_, traE^*^, traG^*^, traK, traL^*^, traM^*^	1	2	2
14	-	ermC, tetK^*^, *aac6-aph2a*	*pre*_pT181_, traE, traG^*^, *traK, traL*	0	2	0
15	ERY	ermB, ermC, ermG^*^, *tetK, aac6-aph2a*	*pre*_pSK41_, *pre*_pT181_, traL^*^	1	1	1
16	ERY	*ermB, ermC, tetK, aac6-aph2a*	*pre*_pSK41_, *nes*_pSK41_, *pre*_pT181_, traE^*^, traG^*^, *traK, traL*	1	0	0
17	ERY	*ermB, ermC, tetK, aac6-aph2a*	*pre*_pSK41_, *nes*_pSK41_, *pre*_pT181_, *traE, traG, traK, traL, traM*	0	1	2
18	–	ermB^*^, *ermC, tetK, aac6-aph2a*	*pre*_pSK41_, *nes*_pSK41_, *pre*_pT181_, *traE, traG, traK, traL*	0	1	0
19	–	*ermC, tetK, aac6-aph2a*	*pre*_pSK41_, *pre*_pT181_, traK^*^, *traM*	0	2	2
20	ERY, GEN	ermB^*^, *ermC, tetK, aac6-aph2a*	*pre*_pSK41_, *pre*_pT181_, traE^*^, traG^*^, traK^*^, *traL, traM*	1	1	2
21	ERY, GEN	ermB^*^, *ermC, tetK*	*pre*_pSK41_, *pre*_pT181_, *traE, traG, traK, traL, traM*	1	1	2
22	–	*ermB, ermC, tetK, aac6-aph2a*	*pre*_pSK41_, *pre*_pT181_, traE^*^, traG^*^, *traL, traM*	1	2	3
23	ERY, GEN	*ermC, aac6-aph2a*	*pre*_pSK41_, *pre*_pT181_, traE^*^, *traG, traL*	0	2	2
24	GEN	ermB^*^, *ermC, aac6-aph2a*	*pre*_pSK41_, *pre*_pT181_,	0	3	2
25	ERY, GEN	*ermB, ermC, tetK, tetM, aac6-aph2a*	*pre*_pSK41_, *pre*_pT181_, traG^*^, traK^*^, *traL, traM*	0	1	3

a *GEN, gentamicin; ERY, erythromycin; TET, tetracycline*.

b *aac6-aph2a, gentamicin; ermB/ermC/ermG, erythromycin; tetK/tetM, tetracycline; vanB, vancomycin resistance genes*.

c *Numbers indicate the number of plasmid bands observed in the 1% agarose gel or in the PFGE*.

d *0, no biofilm-forming capacity; 1, weak biofilm-forming capacity; 2, strong biofilm-forming capacity, 3, very strong biofilm-forming capacity*.

* *Weak signal intensity in the Southern blot*.

When agarose gel electrophoresis was used, it was observed that 44% of the clinical isolates contained at least one plasmid with a size <20 kb (Figure 1 and Table 5). In particular, nine of the isolates contained only one plasmid smaller than 20 kb. Isolate 12 harbored two plasmids smaller than 20 kb, while 4 plasmids of this size were identified in isolate 9.

According to our PFGE data, 84% of the clinical isolates (all except isolates 1, 3, 4, and 16) contained at least one large plasmid (Figure 2 and Table 5): 32% of the isolates (6, 10, 15, 17, 18, 20, 21, and 25) harbored one large plasmid; 32% of the isolates (2, 5, 8, 13, 14, 19, 22, and 23) contained two large plasmids; and 20% of the isolates (7, 9, 11, 12, and 24) harbored three large plasmids.

### All clinical isolates contained antibiotic resistance genes

Eight resistance genes commonly found in staphylococci were investigated by PCR and Southern blotting: genes encoding resistance to erythromycin (*ermB, ermC, ermD, ermG*), tetracycline (*tetK, tetM*), gentamicin (*aac6-aph2a*), and vancomycin (*vanB*). The presence of these genes was tested in our extracted DNA (i.e., putative plasmid DNA) because, initially, we were only interested in the risk of dissemination of antibiotic resistance from these clinical strains through bacterial conjugation.

**Figure 3:**
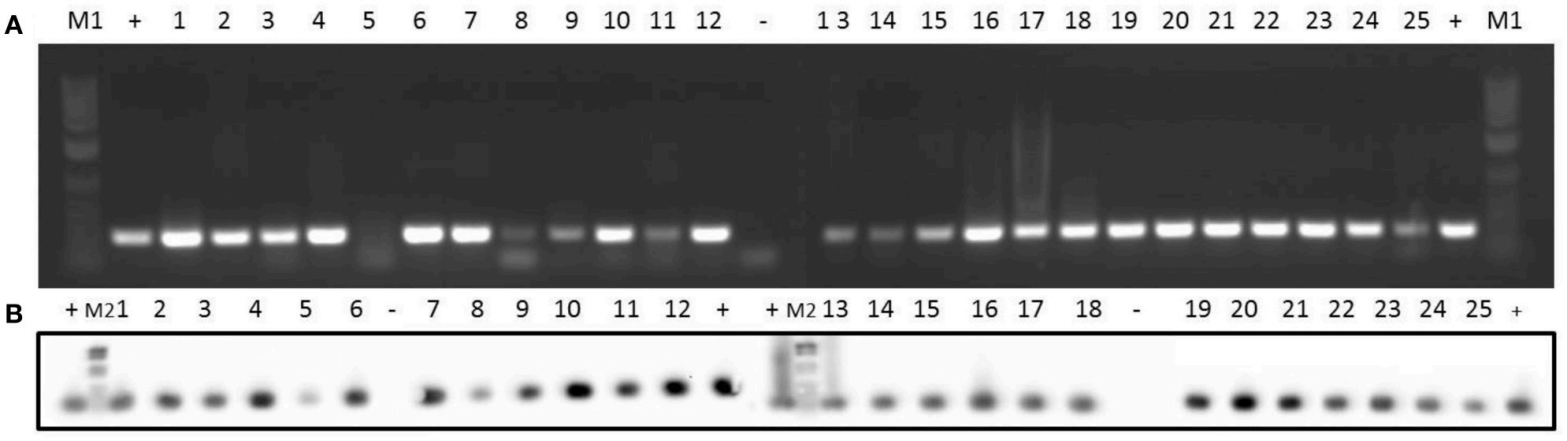
Detection of erythromycin resistance gene *ermC* in 25 clinical isolates by PCR **(A)** and Southern blotting **(B)**. Amplicons of *ermC* (477 bp) were visualized on 1% (w/v) agarose gels. Lanes 1–25: clinical isolates. Lanes +: positive control. Lanes –: negative control. Lanes M1: DNA molecular weight marker 1 kb Plus DNA Ladder. Lanes M2: DNA molecular weight marker VI DIG-labeled.

Concerning erythromycin resistance, 15 of the strains had an erythromycin resistance phenotype (Table 1). Data at the genotype level for the different clinical isolates are shown in Table 5. *ermC* gene was observed in all the isolates (Figure 3), while *ermD* was not detected in any of the isolates (Supplementary Figure 2). Likewise, 72% of the isolates were *ermB*-positive (Supplementary Figure 3), whereas only 8% of the isolates (2 and 15) harbored the *ermG* gene (Supplementary Figure 4).

With respect to tetracycline, only two isolates (9 and 12) were observed to be tetracycline resistant at the phenotype level (Table 1). Regarding this antibiotic, 23 out of 25 isolates contained the *tetK* gene (Supplementary Figure 5), while only isolate 25 harbored the *tetM* gene (Supplementary Figure 6).

Similarly, the 25 isolates were analyzed for the occurrence of the gentamicin resistance *aac6-aph2a* gene. As shown in Supplementary Figure 7, this gene was detected in 88% of the isolates (all the isolates except 9, 11, and 21 showed a positive result for the *aac6-aph2a* gene). However, according to the phenotype (Table 1), only 44% of the isolates showed gentamicin resistance.

**Figure 4:**
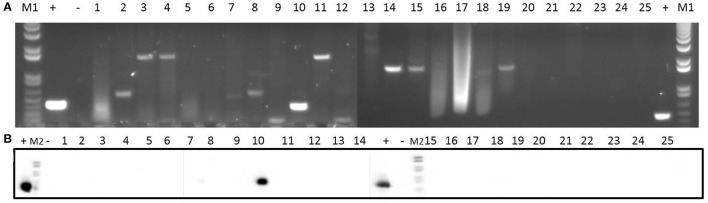
Detection of vancomycin resistance gene *vanB* in 25 clinical isolates by PCR **(A)** and Southern blotting **(B)**. Amplicons of *vanB* (539 bp) were visualized on 1% (w/v) agarose gels. Lanes 1–25: clinical isolates. Lanes +: positive control. Lanes −: negative control. Lanes M1: DNA molecular weight marker 1 kb Plus DNA Ladder. Lanes M2: DNA molecular weight marker VI DIG-labeled.

Finally, regarding vancomycin resistance, all the isolates were phenotypically sensitive to this antibiotic (Table 1). At the genotype level, only isolate 10 proved to be *vanB*-positive (Figure 4).

### All clinical isolates encoded relaxase and/or horizontal transfer genes commonly found in *Staphylococcus* conjugative/mobilizable plasmids

In order to find out whether the abovementioned antibiotic resistance genes were likely to be disseminated via conjugative transfer, we searched for horizontal transfer genes from two common staphylococcal plasmids: (i) conjugative pSK41 and (ii) mobilizable pT181 (Novick, 1989; Berg et al., 1998).

**Figure 5:**
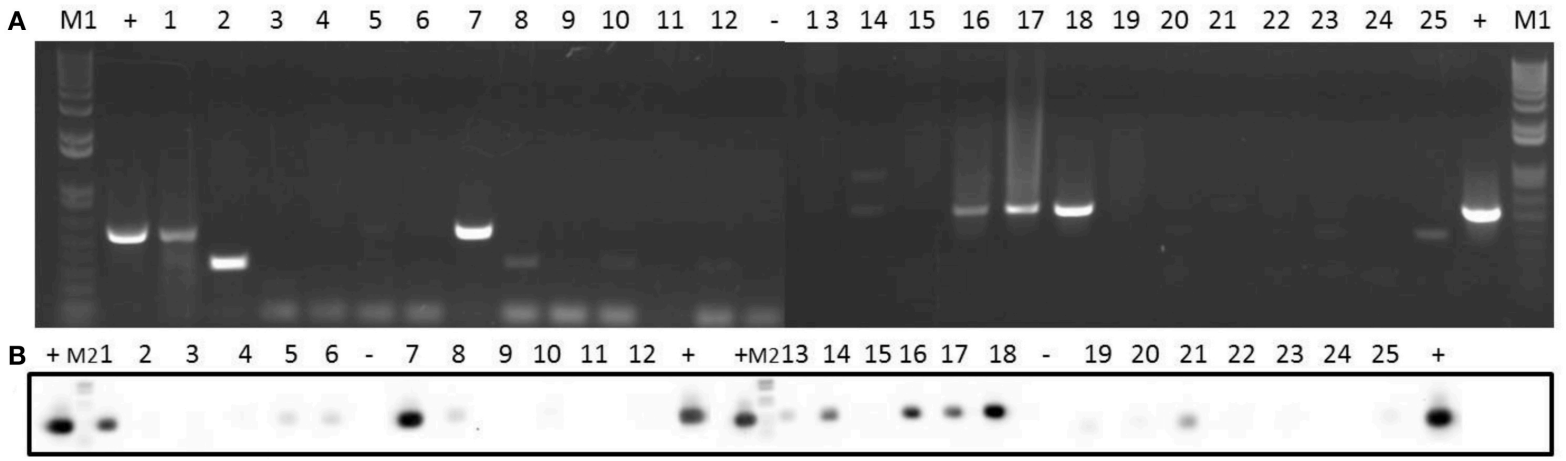
Detection of *traK* gene in 25 clinical isolates by PCR **(A)** and Southern blotting **(B)**. Amplicons of *traK* gene (573 bp) were visualized on 1% (w/v) agarose gels. Lanes 1–25: clinical isolates. Lanes +: positive control. Lanes −: negative control. Lanes M1: DNA molecular weight marker 1 kb Plus DNA Ladder. Lanes M2: DNA molecular weight marker VI DIG-labeled.

In relation to pSK41, the *pre* relaxase gene was found in all the isolates except isolate 14 (Supplementary Figure 8). In addition, isolates 1, 4, 7, 8, 11, 16, 17, and 18 contained the *nes* relaxase gene of pSK41 (Supplementary Figure 9). Five genes (*traE, traG, traK, traL*, and *traM*) from the transfer region of pSK41 were also analyzed: *traE* gene was present in 48% of the isolates (Supplementary Figure 10), *traG* gene was detected in 68% of the isolates (Supplementary Figure 11), *traK* gene was found in 56% of the isolates (Figure 5), and *traL* gene was detected in 88% of the isolates (Supplementary Figure 12). Finally, *traM* gene was found in only 36% of the isolates (Supplementary Figure 13).

**Figure 6:**
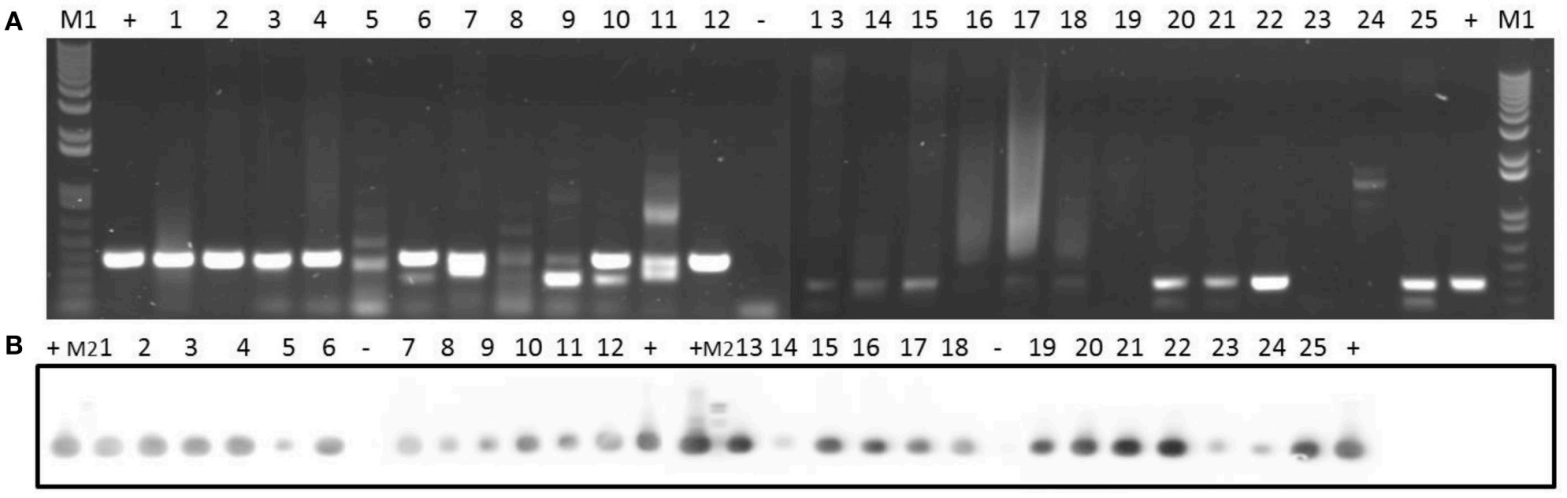
Detection of *pre*_pT181_ gene in 25 clinical isolates by PCR **(A)** and Southern blotting **(B)**. Amplicons of *pre*_pT181_ gene (397 bp) were visualized on 1% (w/v) agarose gels. Lanes 1–25: clinical isolates. Lanes +: positive control. Lanes −: negative control. Lanes M1: DNA molecular weight marker 1 kb Plus DNA Ladder. Lanes M2: DNA molecular weight marker VI DIG-labeled.

In addition, we tested for the presence of the *pre* relaxase gene of the staphylococcal mobilizable plasmid pT181. As shown in Figure 6, this gene was detected in all the clinical isolates.

### Clinical isolates differed in their biofilm-forming capacity

All the clinical strains were isolated from biofilms formed on medical devices such as catheters and prostheses, as well as from ulcer and articular fluids from patients with prostheses (Table 1). In order to confirm their biofilm forming capacity, we used the *in vitro* assay described above (Christensen et al., 1985; Di Rosa et al., 2006).

**Figure 7:**
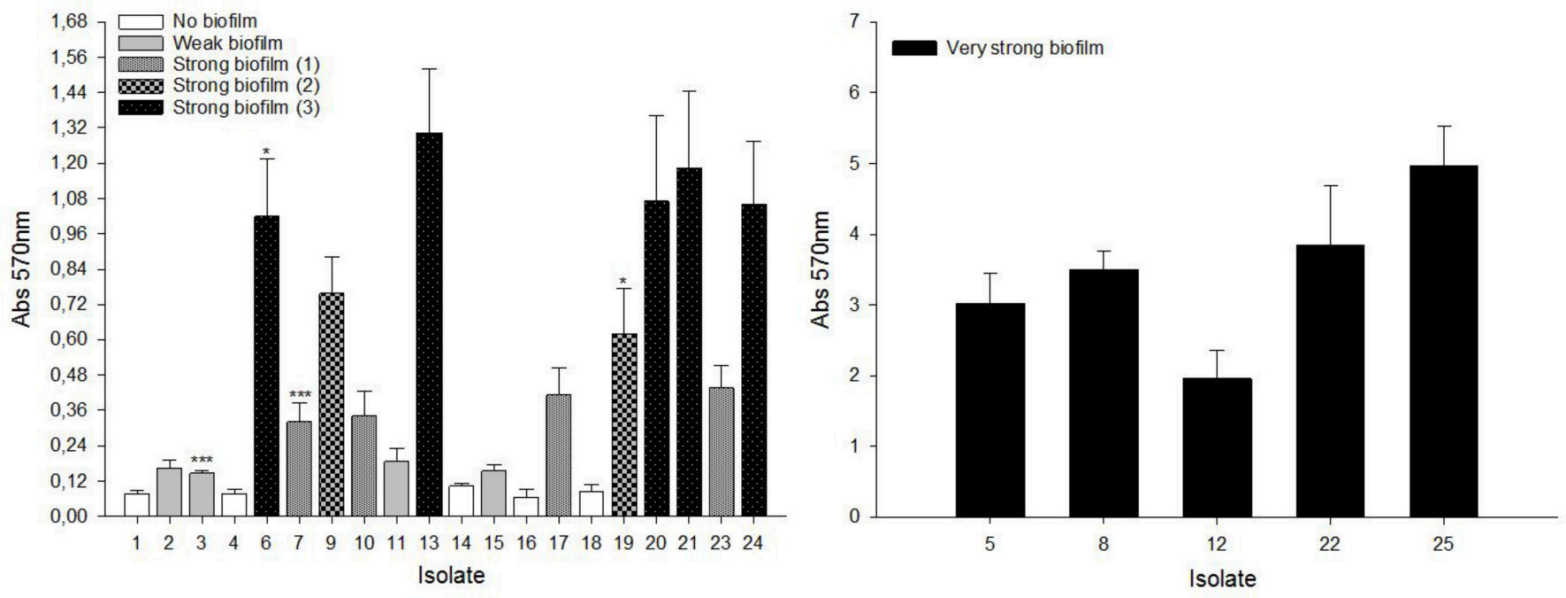
Biofilm-forming capacity of staphylococcal clinical isolates. 96-well flat-bottom polystyrene plates were incubated for 24 h at 37°C without shaking. Cells attached to the wells were stained with 0.1% (w/v) crystal violet. Absorption at 570 nm was measured to quantify biofilm formation. Results are the mean ± SEM of at least 4 independent biological experiments performed in triplicate. To classify the isolates into significant groups, statistical analysis was performed using Student's *t*-test or Mann–Whitney *U*-test (^*^p < 0.05, ^***^p < 0.001).

As shown in Figure 7, isolates were divided into four groups: (i) no biofilm-forming isolates: 1, 4, 14, 16, 18; (ii) weak biofilm-forming isolates: 2, 3, 11, 15; (iii) strong biofilm-forming isolates: 6, 7, 9, 10, 13, 17, 19, 20, 21, 23, 24; and (iv) very strong biofilm-forming isolates: 5, 8, 12, 22, 25. Additionally, our statistical analysis showed that the “strong biofilm-forming” group could be further divided into three different sub-groups with increasing biofilm forming capacity from “strong biofilm-forming (1)” to “strong biofilm-forming (3).” The distribution of isolates in these three sub-groups was as follows: “strong biofilm-forming (1)”: 7, 10, 17, 23; “strong biofilm-forming (2)”: 9, 19; and “strong biofilm-forming (3)”: 6, 13, 20, 21, 24 (Figure 7). Furthermore, the relationship between biofilm-forming capacity and Staphylococcus species was studied. *S. epidermidis* isolates had a significantly higher (*p* < 0.001) biofilm-forming capacity than *S. aureus* isolates.

## Discussion

Staphylococci nosocomial pathogens are frequently involved in biomaterial-associated infections (Pfaller and Herwaldt, 1988; Kloos and Bannerman, 1994; Huebner and Goldmann, 1999; Otto, 2008). The eradication of these biofilm-associated infections with antibiotic treatment is usually impossible without the removal of the medical device (Stewart and Costerton, 2001; Mack et al., 2004; Otto, 2012; Tong et al., 2012). Furthermore, conjugative plasmid-mediated dissemination of antibiotic resistance is favored in bacterial biofilms (Ghigo, 2001; Molin and Tolker-Nielsen, 2003; Reisner et al., 2006; Yang et al., 2008; D'Alvise et al., 2010).

In this work, 25 staphylococcal biofilm-forming clinical isolates were studied. First, plasmids of different sizes were detected. Secondly, antibiotic resistance and transfer genes were detected by PCR and Southern blotting. Finally, the capacity of these isolates to form biofilms *in vitro* was studied.

Fifteen plasmids smaller than 20 kb and 39 plasmids larger than 20 kb were found in 11 (44%) and 21 (84%) isolates, respectively. This higher percentage of isolates with large plasmids, compared to isolates with small plasmids, is in agreement with results obtained by Shearer et al. (2011) who found that 79% of their isolates harbored at least one large (>20 kb) plasmid. According to Smillie et al. (2010), in proteobacteria, 58% of the plasmids larger than 20 kb are mobilizable. Therefore, our results suggest that almost all our *Staphylococcus* clinical isolates could harbor conjugative and/or mobilizable plasmids.

On the other hand, the presence of antibiotic resistance and horizontal transfer genes commonly found staphylococci was investigated. Antibiotic sensitivity tests (to obtain the well-known antibiograms) are the most common method to determine antibiotic resistance of pathogenic bacteria. Nonetheless, the study of antibiotic resistance at the genotype level is crucial to get information on the potential of those bacteria to develop and disseminate resistance against antibiotics (Palmer and Kishony, 2013). Erythromycin, tetracyclines, gentamicin, and vancomycin are the most used antibiotics for the treatment of staphylococcal infections, but resistance genes against these antibiotics have been described in staphylococcal clinical isolates (Duran et al., 2012; Emaneini et al., 2013). Therefore, we searched in our 25 clinical isolates for the presence of genes involved in the resistance to these antibiotics.

Macrolide antibiotics, such as erythromycin, are broad-spectrum antibiotics; relevantly, anti-biofilm activities have been assigned to them (Parra-Ruiz et al., 2012; Zhao et al., 2015). In this work, 60% of the isolates were phenotypically resistant to erythromycin. At the genotype level, different studies have reported the prevalence of the *ermC* gene in staphylococci (Duran et al., 2012; Schiwon et al., 2013); in our study, the *ermC* gene was identified in all the clinical isolates. Although a low prevalence of the *ermB* gene has been described in Staphylococcus (Zmantar et al., 2011), in our study, 72% of the isolates harbored the *ermB* gene.

After penicillin, tetracyclines are the second most widely used group of antibiotics worldwide (van Hoek et al., 2011). Resistance to tetracycline can be encoded in plasmid-located *tet* genes such as *tetK* and *tetL*, or, alternatively, in genes located in the chromosome or transposons such as *tetM* and *tetO* (Emaneini et al., 2013). A high incidence of the *tetK* gene (92%) was observed here, whereas only one isolate contained the *tetM* gene. Several studies have reported the coexistence of both *tetM* and *tetK* genes in staphylococci strains (Duran et al., 2012; Camoez et al., 2013; Emaneini et al., 2013; Schiwon et al., 2013). Here, a disagreement between phenotypic and genotypic data was observed, since only 8% of the isolates were phenotypically resistant to tetracycline.

Aminoglycosides, such as gentamicin, are broad-spectrum antibiotics used against *S. aureus* infections. Aminoglycoside modifying enzymes (AME) are used by bacteria to abolish the effect of these antibiotics. In *S. aureus* strains, one of the most common genes encoding AME is the *aac6-aph2a* gene (Emaneini et al., 2013). In our study, 88% of the isolates contained this gene, in agreement with other studies on staphylococcal isolates (Duran et al., 2012; Emaneini et al., 2013). In terms of the phenotype, 44% of the isolates showed resistance to gentamicin (Table 1). Similar discrepancies between phenotypic and genotypic results have been observed by other authors (Duran et al., 2012; Emaneini et al., 2013).

The lack of correlation between resistance phenotypic and genotypic data could be due to mutations in genes resulting in non-functional proteins, as well as to the lack of gene expression (Martineau et al., 2000). Also, methods to detect antibiotic resistance phenotype are influenced by technical variables such as temperature, incubation time, inoculum density and so on (Baddour et al., 2007). Likewise, the pattern of negative resistance phenotype together with a positive resistance genotype can be due to the presence of pseudogenes (Davis et al., 2011). As a consequence, it is essential to take this fact into account because it indicates that bacteria have the potential to be resistant to more antibiotics than those shown phenotypically.

Vancomycin has been used to treat staphylococcal infections, mainly methicillin resistant *S. aureus* (Huebner and Goldmann, 1999). In the late 1980s, the emergence of vancomycin resistance was reported for the first time (van Hoek et al., 2011). One of the genes responsible for vancomycin resistance is the *vanB* gene (van Hoek et al., 2011), which was only found in isolate 10. This low incidence of the *vanB* gene, together with the fact that all isolates were phenotypically sensitive to vancomycin (Table 1), suggest that (i) vancomycin is still one of the best options for the treatment of staphylococcal infections and (ii) it should be then used judiciously.

Concerning the presence of horizontal transfer genes, plasmid pSK41 is a prototypical multiresistance plasmid of 46 kb from *S. aureus* (Berg et al., 1998). Therefore, we searched for *pre*_pSK41_ and *nes*_pSK41_ genes, as well as for five different *tra* genes involved in the conjugative transfer of plasmid pSK41, in our clinical isolates. Although all the isolates, except for one, contained the *pre*_pSK41_ gene, only 32% of the isolates harbored the *nes*_pSK41_ gene. In addition, 20% of the isolates contained the five *tra* genes tested here. According to these results, and taking into account that isolates 11 and 17 harbored plasmids of around 46 kb, we speculate that these strains could contain pSK41-type plasmids. Other studies have identified plasmids of the pSK41 family in geographically diverse isolates of both *S. aureus* and CoNS (Berg et al., 1998). The coexistence of *pre*_pSK41_ and *nes*_pSK41_ genes, together with *tra* genes, in some of our isolates, points to a risk of dissemination of resistance traits.

pT181 plasmid is also common among staphylococci (Khan and Novick, 1983; Novick, 1989). Then, we tested for the presence of the relaxase *pre*_pT181_ gene responsible for pT181 mobilization. All isolates harbored the *pre*_pT181_ relaxase gene, suggesting that pT181-type plasmids could be present in all of the samples. pT181 is a low copy number plasmid and then, not surprisingly, we could not detect it in the electrophoretic gels; however, it may be present at undetectable amounts in some strains. This is of great concern especially in those strains where potentially conjugative plasmids that could mobilize these small plasmids are present.

Finally, in general, *S. epidermidis* isolates have shown a higher biofilm-forming capacity than *S. aureus* isolates Although, the 25 isolates studied here were obtained from biofilms present in the clinical environment, some of the *S. aureus* isolates were unable to form biofilms under our experimental conditions. This is probably because biofilms in clinical conditions take longer times to form, in comparison to the standardized *in vitro* method used here, in which 24 h was the biofilm-forming time.

The fact that our clinical isolates contained both antibiotic resistance and horizontal transfer genes, as well as conjugative and/or mobilizable plasmids, suggest the possibility of their disseminating antibiotic resistance to other bacteria. Here, it must be stated that, due to the abovementioned presence of chromosomal DNA in our extracted DNA samples, we cannot rule out the possibility that the antibiotic resistance genes identified here were encoded in the chromosomal DNA. However, as reflected in Figures 1, 2, the majority of the extracted DNA corresponds to plasmid DNA. For example, the high incidence of the plasmid-encoded *tetK* gene in our isolates could support this fact. In any case, genes encoded in the chromosome can also be mobilized between bacterial cells. For instance, transposons can mobilize chromosomal genes by jumping into plasmids or phages which can then be transferred into other cells (Frost et al., 2014). On the other hand, the conjugation process can also occur via chromosomally integrated conjugative elements, such as conjugative transposons. Integrated conjugative elements are known to encode proteins that facilitate their own transfer and sometimes the transfer of other cellular DNA from the donor (Frost et al., 2014). Indeed, as reported by Wilkins and Frost (2001), many plasmids and integrated conjugative elements can effect the transfer of chromosomal DNA. Then, if some of the antibiotic resistance genes identified here were encoded in the chromosomal DNA present in some of our samples, the risk of transfer to other bacterial cells would still exist, although *a priori* lower than if they were encoded in the observed plasmids.

Recent studies underline the importance of collecting more epidemiological data on antibiotic resistance, in order to design novel control strategies for this growing global health problem (Frieri et al., 2016). The isolation and molecular characterization of plasmids from nosocomial pathogens will provide valuable information in the search for new strategies to control the dissemination of antibiotic resistance among clinical pathogens.

## Author contributions

SÁ and IÁR: Acquisition of the data, writing and revision of the content, approval of the last version of the work. OG: Revision of the content, approval of the last version of the work. CG: Writing and revision of the content, approval of the last version and ensuring accuracy and integrity of the work. EG: Design of the work, revision of the content, approval of the last version, and ensuring accuracy and integrity of the work. IA: Design of the work and the acquisition of the data, writing, and revision of the content, approval of the last version and ensuring accuracy and integrity of the work.

### Conflict of interest statement

The authors declare that the research was conducted in the absence of any commercial or financial relationships that could be construed as a potential conflict of interest. The reviewer GDS and handling Editor declared their shared affiliation.
