# Supplementary material for: Biofilm-Forming Clinical Staphylococcus Isolates Harbor Horizontal Transfer and Antibiotic Resistance Genes
Source: Front Microbiol. 2017 Oct 16;8:2018. doi: 10.3389/fmicb.2017.02018 (PMC5650641; doi:10.3389/fmicb.2017.02018)
Supplement: Supplementary file 1 [file Table1.DOCX]

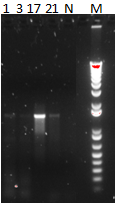


**Supplementary Figure 1. Analysis of PCR samples and template DNAs.** 16S rRNA was amplified by PCR as explained in Broszat *et al*., 2014. The obtained samples were analyzed by 1% (w/v) agarose gel electrophoresis in 1 X TAE buffer. Numbers correspond to the isolate number. N: Negative control. M: DNA molecular weight marker 1 kb Plus DNA Ladder.


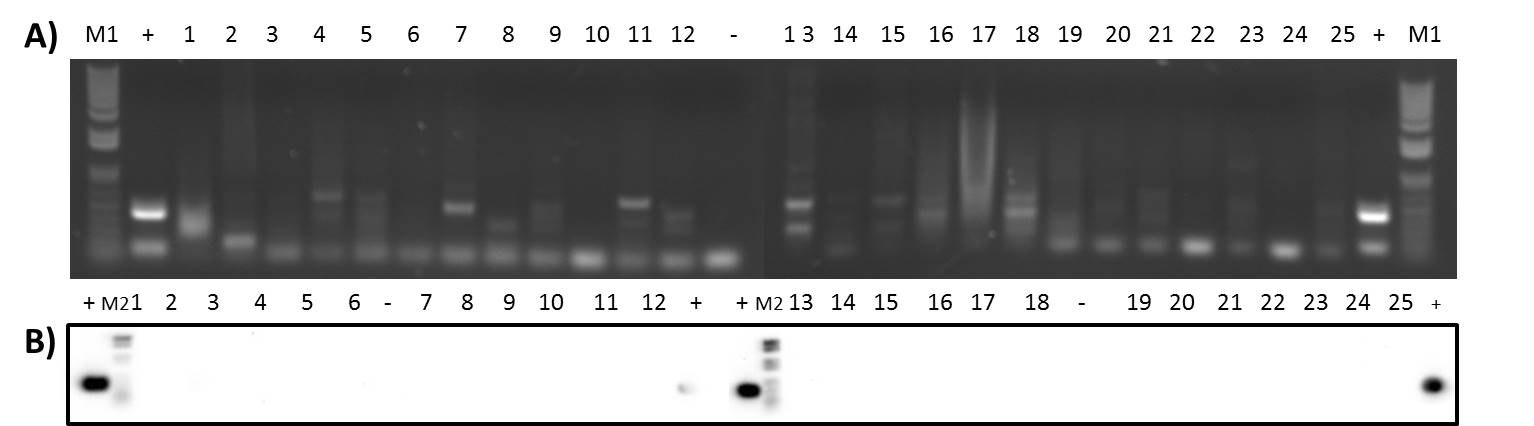


**Supplementary Figure 2. Detection of erythromycin resistance gene *ermD* in 25 clinical isolates by PCR (A) and Southern blotting (B)**. Amplicons of *ermD* (463 bp) were visualized on 1% (w/v) agarose gels. Lanes 1-25: clinical isolates. Lanes +: positive control. Lanes -: negative control. Lanes M1: DNA molecular weight marker 1 kb Plus DNA Ladder. Lanes M2: DNA molecular weight marker VI DIG-labeled.


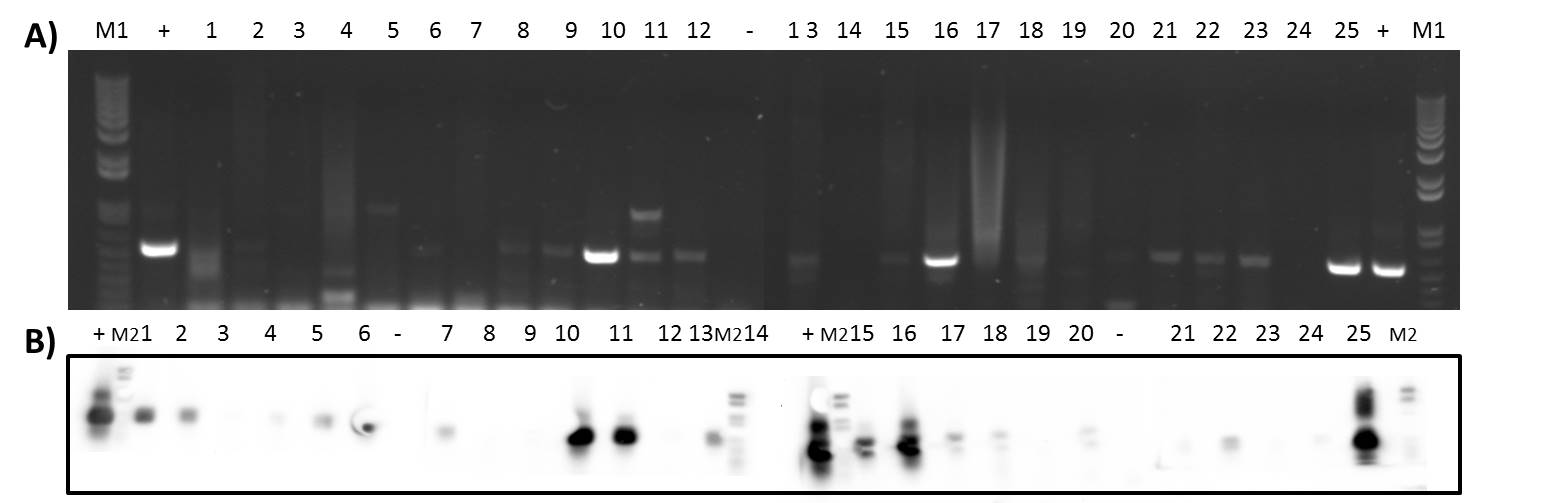


**Supplementary Figure 3. Detection of erythromycin resistance gene *ermB* in 25 clinical isolates by PCR (A) and Southern blotting (B)**. Amplicons of *ermB* (572 bp) were visualized on 1% (w/v) agarose gels. Lanes 1-25: clinical isolates. Lanes +: positive control. Lanes -: negative control. Lanes M1: DNA molecular weight marker 1 kb Plus DNA Ladder. Lanes M2: DNA molecular weight marker VI DIG-labeled.


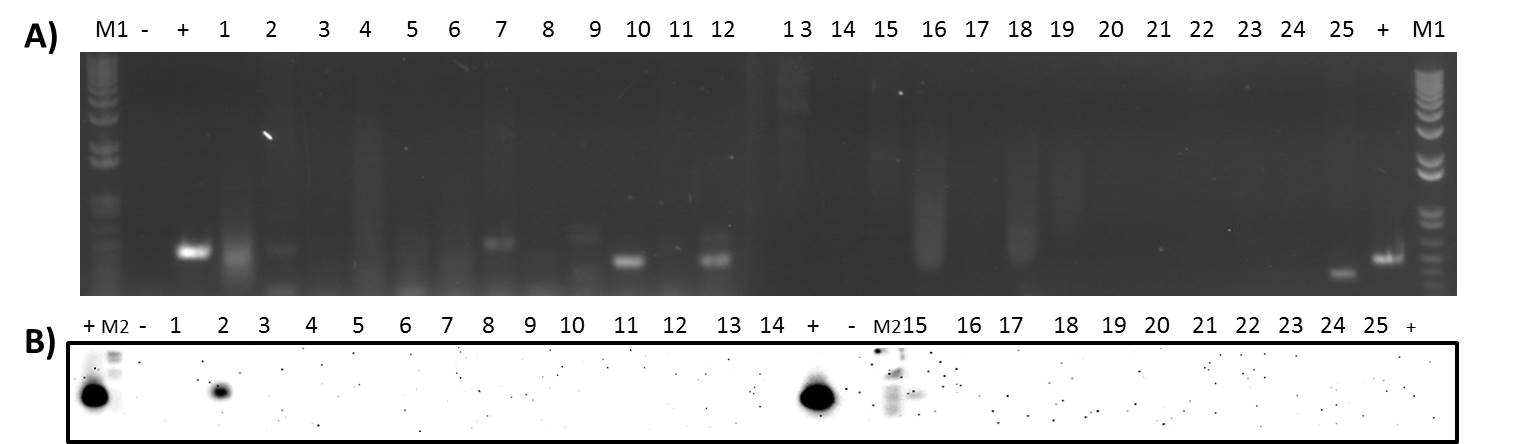


**Supplementary Figure 4. Detection of erythromycin resistance gene *ermG* in 25 clinical isolates by PCR (A) and Southern blotting (B)**. Amplicons of *ermG* (483 bp) were visualized on 1% (w/v) agarose gels. Lanes 1-25: clinical isolates. Lanes +: positive control. Lanes -: negative control. Lanes M1: DNA molecular weight marker 1 kb Plus DNA Ladder. Lanes M2: DNA molecular weight marker VI DIG-labeled.


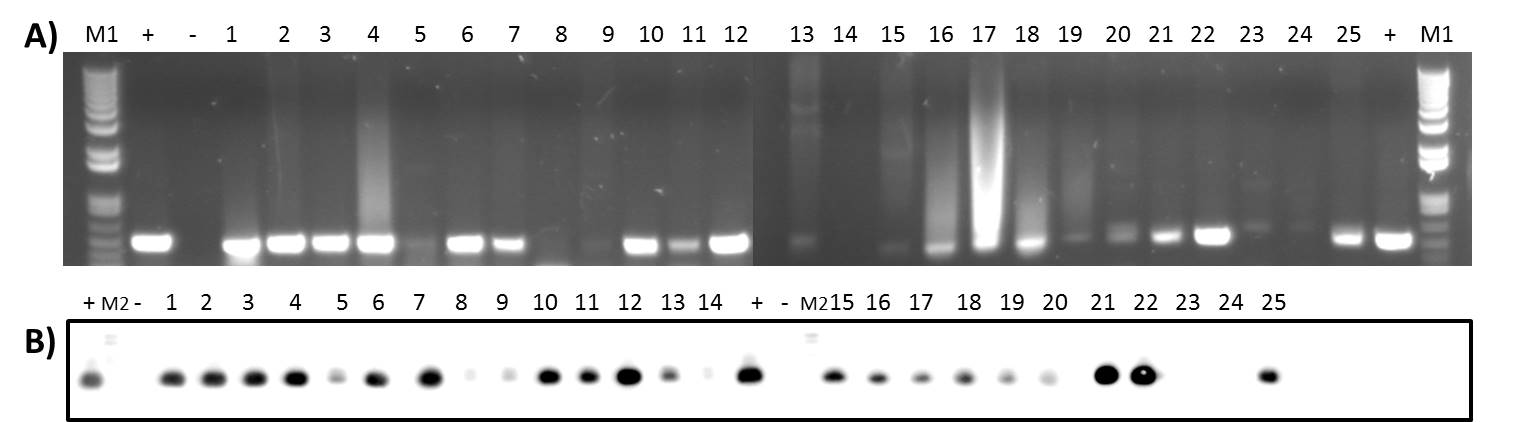


**Supplementary Figure 5. Detection of tetracycline resistance gene *tetK* in 25 clinical isolates by PCR (A) and Southern blotting (B)**. Amplicons of *tetK* (483 bp) were visualized on 1% (w/v) agarose gels. Lanes 1-25: clinical isolates. Lanes +: positive control. Lanes -: negative control. Lanes M1: DNA molecular weight marker 1 kb Plus DNA Ladder. Lanes M2: DNA molecular weight marker VI DIG-labeled.


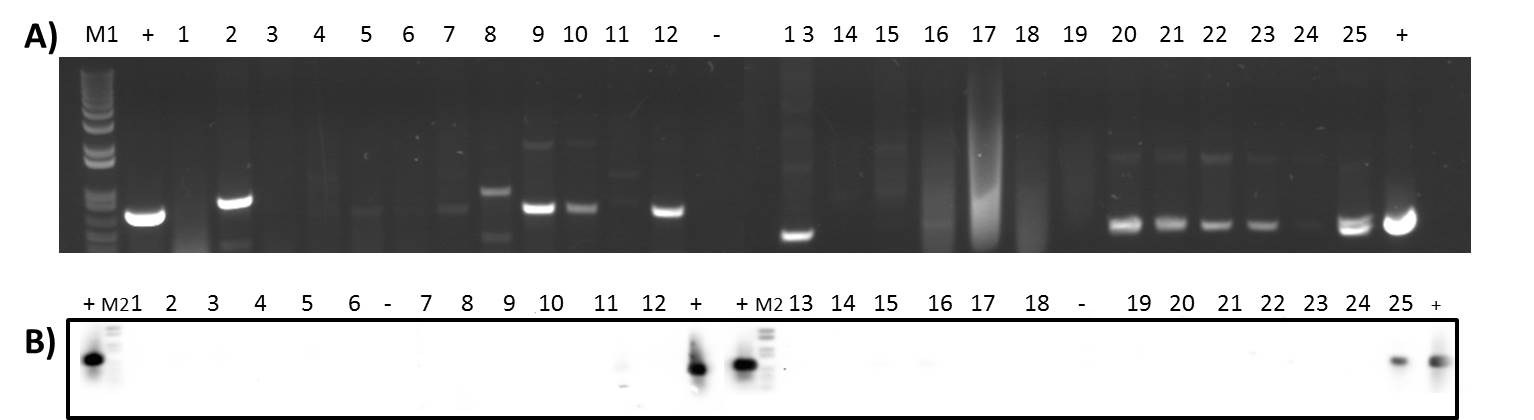


**Supplementary Figure 6. Detection of tetracycline resistance gene *tetM* in 25 clinical isolates by PCR (A) and Southern blotting (B)**. Amplicons of *tetM* (739 bp) were visualised on 1% (w/v) agarose gels. Lanes 1-25: clinical isolates. Lanes +: positive control. Lanes -: negative control. Lanes M1: DNA molecular weight marker 1 kb Plus DNA Ladder. Lanes M2: DNA molecular weight marker VI DIG-labelled.


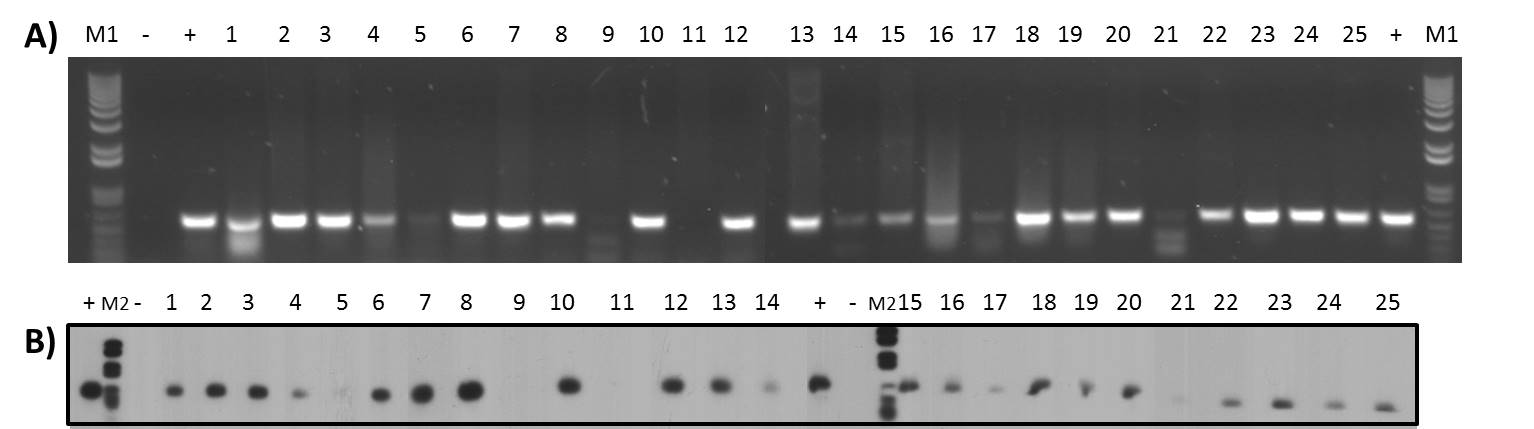


**Supplementary Figure 7. Detection of gentamicin resistance gene *aac6-aph2a* in 25 clinical isolates by PCR (A) and Southern blotting (B)**. Amplicons of *aac6-aph2a* gene (610 bp) were visualized on 1% (w/v) agarose gels. Lanes 1-25: clinical isolates. Lanes +: positive control. Lanes -: negative control. Lanes M1: DNA molecular weight marker 1 kb Plus DNA Ladder. Lanes M2: DNA molecular weight marker VI DIG-labeled.


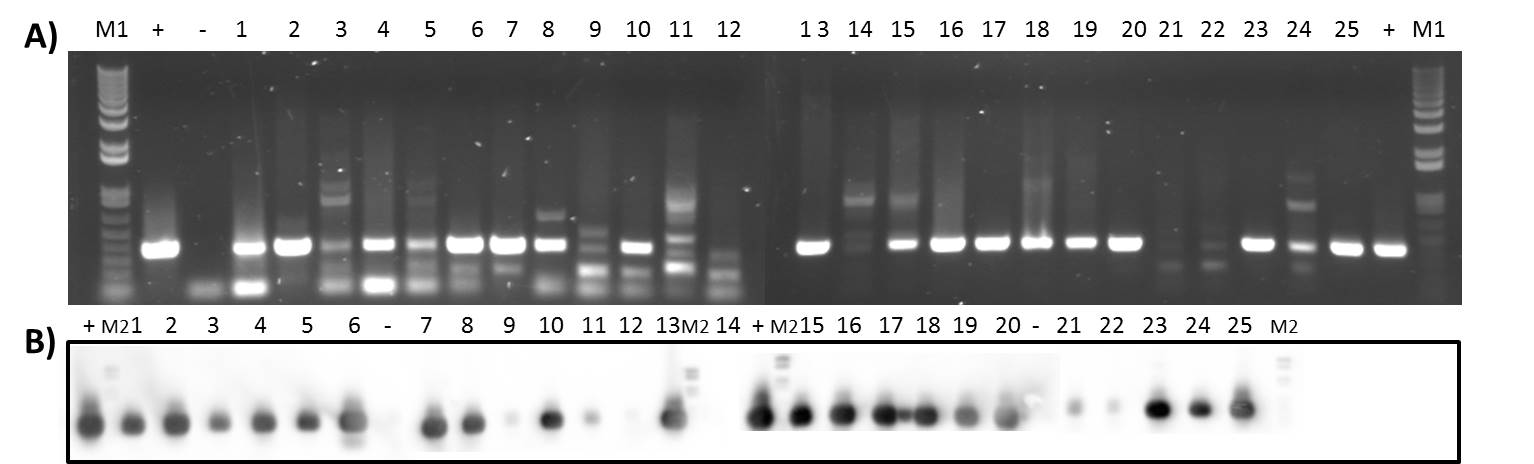


**Supplementary Figure 8. Detection of *pre*_pSK41_ gene in 25 clinical isolates by PCR (A) and Southern blotting (B)**. Amplicons of *pre*_pSK41_ (395 bp) were visualized on 1% (w/v) agarose gels. Lanes 1-25: clinical isolates. Lanes +: positive control. Lanes -: negative control. Lanes M1: DNA molecular weight marker 1 kb Plus DNA Ladder. Lanes M2: DNA molecular weight marker VI DIG-labeled.


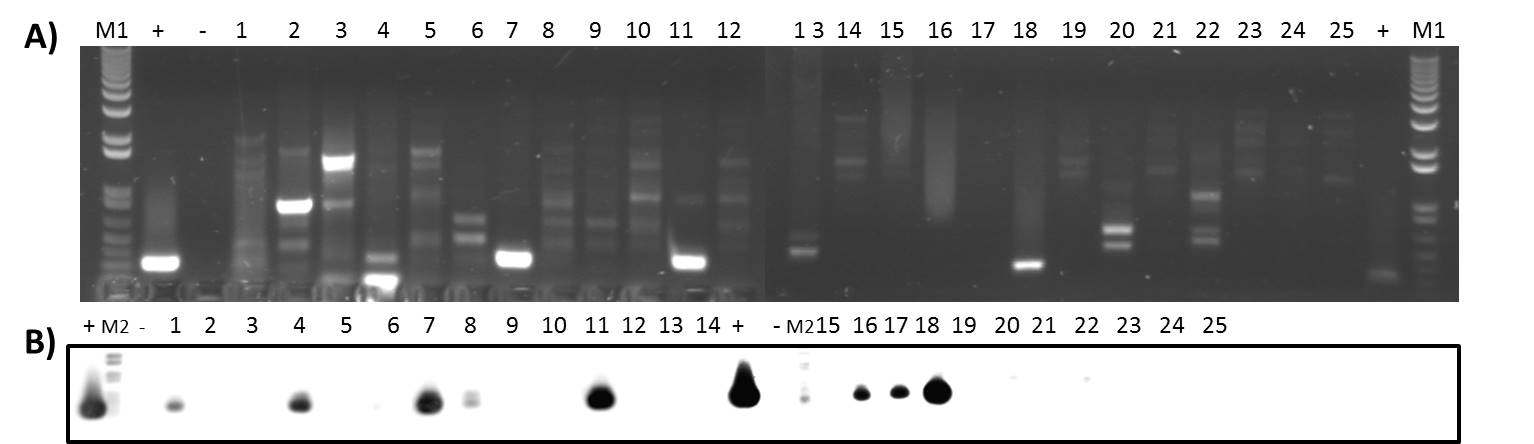


**Supplementary Figure 9. Detection of *nes*_pSK41_ gene in 25 clinical isolates by PCR (A) and Southern blotting (B)**. Amplicons of *nes*_pSK41_ (328 bp) were visualized on 1% (w/v) agarose gels. Lanes 1-25: clinical isolates. Lanes +: positive control. Lanes -: negative control. Lanes M1: DNA molecular weight marker 1 kb Plus DNA Ladder. Lanes M2: DNA molecular weight marker VI DIG-labeled.


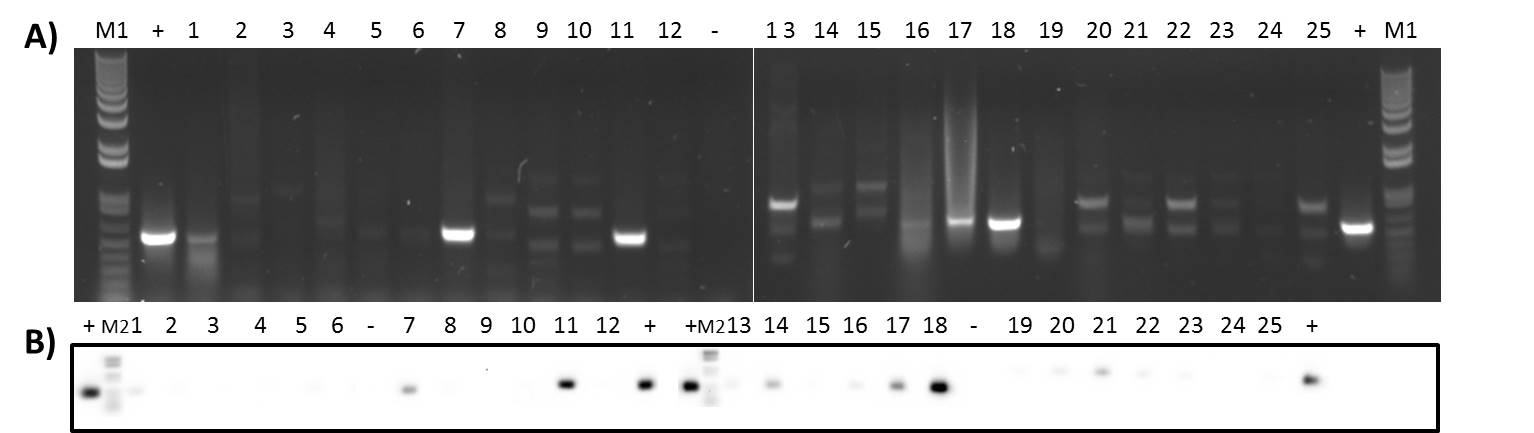


**Supplementary Figure 10. Detection of *traE* gene in 25 clinical isolates by PCR (A) and Southern blotting (B)**. Amplicons of *traE* (626 bp) were visualized on 1% (w/v) agarose gels. Lanes 1-25: clinical isolates. Lanes +: positive control. Lanes -: negative control. Lanes M1: DNA molecular weight marker 1 kb Plus DNA Ladder. Lanes M2: DNA molecular weight marker VI DIG-labeled.


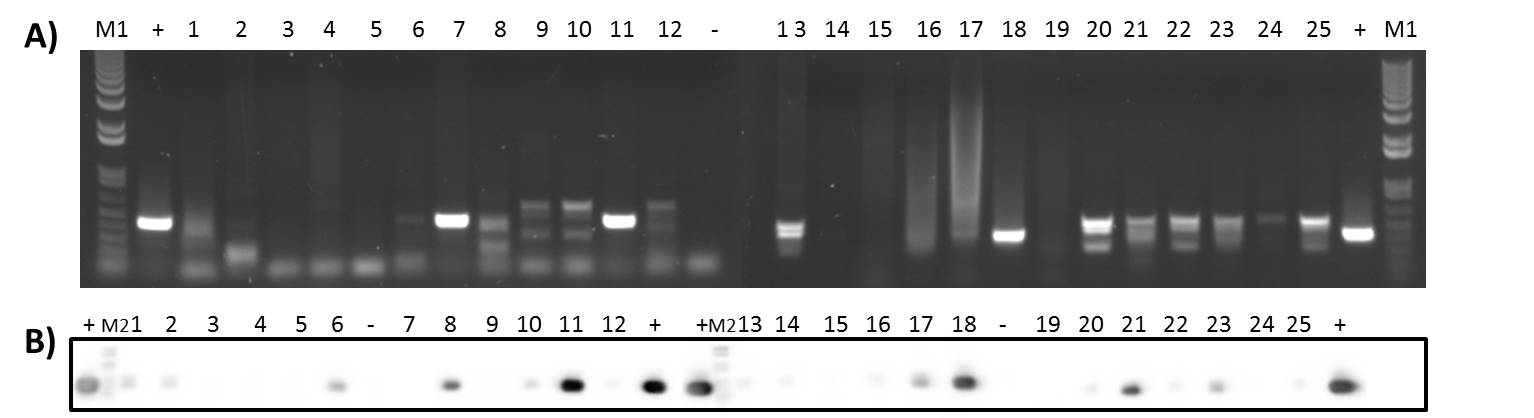


**Supplementary Figure 11. Detection of *traG* gene in 25 clinical isolates by PCR (A) and Southern blotting (B)**. Amplicons of *traG* (457 bp) were visualized on 1% (w/v) agarose gels. Lanes 1-25: clinical isolates. Lanes +: positive control. Lanes -: negative control. Lanes M1: DNA molecular weight marker 1 kb Plus DNA Ladder. Lanes M2: DNA molecular weight marker VI DIG-labeled.


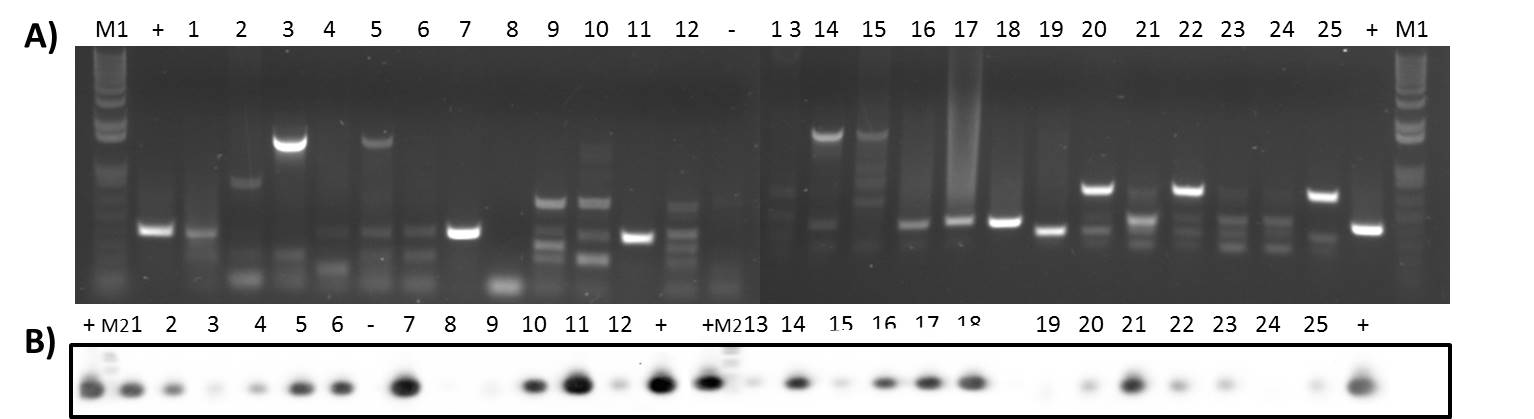


**Supplementary Figure 12. Detection of *traL* gene in 25 clinical isolates by PCR (A) and Southern blotting (B)**. Amplicons of *traL* (407 bp) were visualized on 1% (w/v) agarose gels. Lanes 1-25: clinical isolates. Lanes +: positive control. Lanes -: negative control. Lanes M1: DNA molecular weight marker 1 kb Plus DNA Ladder. Lanes M2: DNA molecular weight marker VI DIG-labeled.


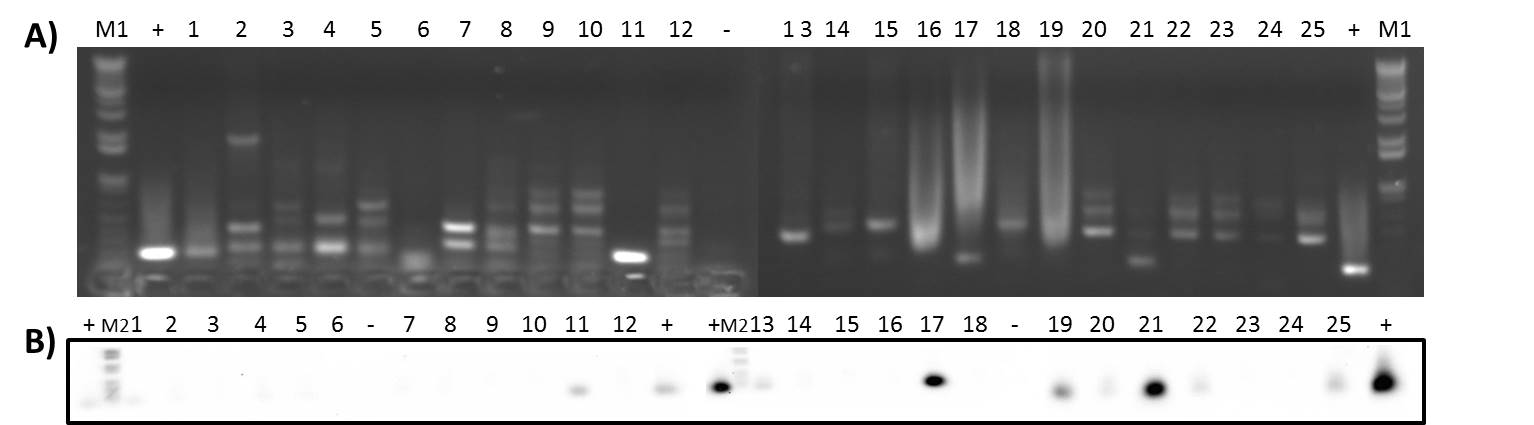


**Supplementary Figure 13. Detection of *traM* gene in 25 clinical isolates by PCR (A) and Southern blotting (B)**. Amplicons of *traM* (200 bp) were visualized on 1% (w/v) agarose gels. Lanes 1-25: clinical isolates. Lanes +: positive control. Lanes -: negative control. Lanes M1: DNA molecular weight marker 1 kb Plus DNA Ladder. Lanes M2: DNA molecular weight marker VI DIG-labeled.
